# Targeted Therapies for Melanoma Brain Metastases

**DOI:** 10.1007/s11940-017-0449-2

**Published:** 2017-04-03

**Authors:** Anna S. Berghoff, Matthias Preusser

**Affiliations:** Department of Medicine I and Comprehensive Cancer Center CNS Unit (CCC-CNS), Medical University of Vienna, Waehringer Guertel 18-20, 1090 Vienna, Austria

**Keywords:** Melanoma, Brain metastases, BRAF inhibitor, Immune checkpoint inhibitor

## Abstract

Brain metastases are a major clinical challenge occurring in up to 60% of patients suffering from metastatic melanoma. They cause significant clinical symptoms and impair the overall survival prognosis. The introduction of targeted therapies including BRAF and MEK inhibitors as well as CTLA-4 and PD-1 axis targeting immune checkpoint inhibitors have dramatically improved the treatment and prognosis of patients with extracranial metastatic melanoma. Although, similar response rates for extra- and intracranial metastases have been reported, only few data from brain metastasis specific trails are available so far. The following review will provide an overview on the currently available data on targeted therapies, remaining questions and the most important side effects in the special clinical situation of melanoma brain metastases.

## Introduction

Brain metastases (BM) are a clinical challenge in the treatment of melanoma patients. Up to 60% of patients suffering from metastatic melanoma develop this life treating and quality of life impairing complication during their course of disease [2]. Melanoma has a particularly high risk to spread to the brain compared to other frequent sources of BM like lung and breast cancer. The median survival after diagnosis of symptomatic BM traditionally ranged from 3.4 to 13.2 months [65].

New targeted treatment modalities, including BRAF and MEK inhibitors as well as immune checkpoint inhibitors, have revolutionized the treatment of metastatic melanoma patients in the last decade [40, 71]. Effective, rapid responses to BRAF inhibitors and long lasting efficacy of immune checkpoint inhibitors have entered the everyday practice. However, all data on the natural course of melanoma brain metastases remains from the era before the availability of targeted therapies. Therefore the course of disease may have significantly changed as, for example, Trastuzumab has significantly changed treatment and prognosis of metastatic breast cancer [32]. However, to this date, the incidence of BM has not decreased since the introduction of the new generation targeted therapies in metastatic melanoma. Additionally, BM are still frequently the first site of progression, making BM treatment a particularly important aspect of melanoma therapy [17, 38]. Approximately one third of melanoma BM patients will die from BM progression, while another third dies from the combined progression of intra- and extracranial metastases [10]. Therefore, treatment strategies have to carefully address this specific progression pattern and combine effective intra- and extracranial treatment approaches.

Carefully designed trials specially concentrating on brain metastatic endpoints are therefore urgently needed. Here, especial emphasis should be given to intracranial response and the potential to prevent symptomatic brain metastases [50]. Further, brain metastasis-specific endpoints like survival without neurological deterioration should be considered and investigated in order to improve the therapy sequence for patients suffering from melanoma brain metastases [50]. A further specific challenge in the treatment of melanoma BM is the relative radio-resistance of melanoma. Therefore, systemic therapies are applied more frequently in oligo- to asymptomatic BM patients as first line treatment approach than in most other BM causing entities [1, 43]. The following review will provide an overview on the available targeted therapies in melanoma and their efficacy in the special context of BM.

## BRAF tyrosine kinase inhibitors

Activating mutations of the *v-Raf murine sarcoma viral oncogene homolog B (BRAF)* gene occur in up to 50% of melanoma patients, with a higher frequency among very young patients. Here, the V600E point mutation with substitution of valine to glutamate at codon 600 is the most frequently observed mutation (>80%) followed by the V600K mutation (14%) [36]. The presence of a BRAF mutation is most likely not associated with an increased risk to develop BM [36]. BRAF tyrosine kinase inhibitors like Vemurafenib and Dabrafenib effectively inhibit the downstream activated MAPK (mitogen-activated protein kinase) pathway and have shown improved disease control rates (CR + PR + SD) of up to 92%, significantly prolonged progression and overall survival compared to chemotherapy in extracranial metastatic melanoma [23, 42].

### Predictive biomarkers

The presence of the V600E and at a lesser extend the V600K BRAF mutation is highly predictive for the response to BRAF inhibitors. The most frequently occurring point mutation V600E can reliable detected with the mutation specific antibody VE1 [12]. However, DNA-based methods like gene sequencing or the COBAS test can also identify less frequently occurring point mutations. Therefore, the sequence of using immunohistochemistry (VE1 antibody) as a screening method followed by a DNA-based method in case of a negative result to rule out rare mutation types is currently proposed for routine clinical practice [51]. Importantly, the BRAF mutation presents with a high concordance between metastases from different sites if using the V600E BRAF mutation specific antibody VE1 [11, 28].

### Clinical efficacy of BRAF inhibitors in melanoma brain metastases

Initial licensing phase III trials excluded patients with active or untreated melanoma BM. However, subsequent BM specific trials have shown similar intra- and extracranial response rates for BRAF inhibitor monotherapy. The BREAK-MB phase II study was the first to specifically address the efficacy of BRAF inhibition in patients with BRAF mutation harboring brain metastatic melanoma [34]. Overall, 172 melanoma BM patients without (*n* = 89; cohort A) or with (*n* = 83; cohort B) prior local BM treatment were included. Intra- (cohort A: 81.1%, cohort B: 89.2%) and extracranial (cohort A: 79.7%, cohort B: 83.1%) disease control rate as well as overall survival (cohort A: 33.1 weeks, cohort B: 31.4 weeks) was impressive and similar in both groups indicating that efficacy of BRAF inhibition is also given in the context of BM recurrence (Table 1). A phase II study of Vemurafenib in a cohort of patients with preciously untreated (*n* = 90; cohort 1) and previously treated (*n* = 56; cohort 2) melanoma BM presented with a very similar outcome (Table 1) [41••]. Intra- (cohort 1: 18%, cohort 2: 20%) and extracranial (cohort 1: 33%, cohort 2: 23%) response rate was comparable as well as overall survival (cohort 1: 8.9 months, cohort 2: 9.6 months), although numerically less impressive compared to the responses observed for Dabrafenib.

**Table 1:** Selected trials on targeted therapies in melanoma brain metastases

Study	Type of trial	Study population	Targeted therapy	Intracranial disease control rate (SD + PR + CR)	Extracranial disease control rate (SD + PR + CR)	OS
BREAK-MB—Long et al.	Phase II	Cohort A: Newly diagnosed BM (*n* = 89) Cohort B: previously treated BM (*n* = 83) Total: *n* = 172	Dabrafenib	BRAF V600E mutant: Cohort A: 81.1% Cohort B: 89.2% BRAF V600K mutant: Cohort A: 6.7% Cohort B: 22.2%	BRAF V600E mutant: Cohort A: 79.7% Cohort B: 8.1% BRAFV600K mutant: Cohort A: 46.7% Cohort B: 50.0%	BRAF V600E mutant: Cohort A: 33.1 weeks Cohort B: 31.4 weeks BRAF V600K mutant: Cohort A: 16.3 weeks Cohort B: 21.9 weeks
McArthur et al.	Phase II	Cohort 1: Newly diagnosed BM (*n* = 90) Cohort 2: previously treated BM (*n* = 56) Total: *n* = 146	Vemurafenib	Cohort 1: 18% Cohort 2: 20%	Cohort 1: 33% Cohort 2: 23%	Cohort 1: 8.9 months Cohort 2: 9.6 months
Margolin et al.	Phase II	Cohort A: asymptomatic BM (*n* = 51) Cohort B: symptomatic BM (*n* = 21)	Ipilimumab	Cohort A: 25% Cohort B: 10%	Cohort A: 33% Cohort B: 10%	Cohort A: 7 months Cohort B: 3.7 months
Goldberg et al.	Phase II	Untreated melanoma (*n* = 18) or non-small cell lung cancer BM	Pembrolizumab	Melanoma cohort (*n* = 18): 42%	Melanoma cohort (*n* = 18): 50%	Melanoma cohort (*n* = 18): median OS not reached after median follow-up of 11.6 months

No head to head comparisons answers the question whether to use Vemurafenib or Dabrafenib upon the occurrence of BM. Preclinical data suggest a better brain distribution of Dabrafenib [44, 63]. Currently, a clinical trial tests the accumulation nucleotide-labeled Dabrafenib in established BM as a new method to predict treatment response (NCT02700763).

Unfortunately, the response to BRAF inhibitors is limited to few months as almost all patients present with a disease recurrence within 12 months [35]. A mixed response can be frequently observed shortly before the recurrence, with still stable extracranial metastases but fast growing and multiple new BM [48]. Interestingly, the BM progression presents with a particular pattern of multiple disseminated lesions, which might reflect an invasive or migratory tumor cell phenotype [22].

### Combination of BRAF and MEK inhibitor

As outlined, although initially highly active, almost all patients developed resistance to BRAF inhibitors alone within 6–7 months most likely trough mutations resulting in MAPK reactivation [56]. In line, combination of BRAF and MEK inhibition, which inhibits the downstream activated MAPK pathways from different sites, resulted in a significantly prolonged progression free survival in extracranial metastatic melanoma [30, 33]. The MEK inhibitor trametinib is subject to P-glycoprotein efflux pumps, which has been shown to limit its efficacy in experimental BM models [68]. Interestingly, a lack of P-glycoprotein in human melanoma BM was observed, encouraging the further clinical investigation [55]. Case series suggest that the combination might also be safe and effective in BM patients, however definitive conclusion can only be drawn from the currently recruiting clinical trials (Table 2) [47•].

**Table 2:** Selected on-going trial in melanoma brain metastases

Study	Type of trial	Targeted therapy	Combination with radiotherapy	Intervention	Primary endpoint
BRAF and MEK inhibitor therapy					
NCT02537600	Phase II	Vemurafenib + combimetinib	None	Combination of vemurafenib + combimetinib in symptomatic and asymptomatic BM patients	Intracranial response rate
NCT02974803	Phase II	Dabrafenib + trametinib	Stereotactic radiosurgery	Concurrent dabrafenib + trametinib with stereotactic radiosurgery in active BM	Intracranial objective response rate
Immune checkpoint inhibitor monotherapy					
NCT02085070	Phase II	Pembrolizumab	None	Pembrolizumab monotherapy in asymptomatic patients	Objective response rate
NCT02621515	Phase II	Nivolumab	None	Nivolumab monotherapy in BM patients without or stable steroid dosage	Intracranial best overall response
Combination of two immune checkpoint inhibitors					
NCT02374242	Phase II	Ipilimumab + nivolumab	None	Nivolumab vs. nivolumab + ipilimumab in patients with asymptomatic BM	Intracranial response rate
NCT02460068	Phase III	Ipilimumab + nivolumab	None	Fotemustin + ipilimumab vs. Ipilimumab + nivolumab vs. fotemustin alone in patients with asymptomatic BM	Overall survival
NCT02320058	Phase II	Ipilimumab + nivolumab		Ipilimumab + nivolumab followed by nivolumab monotherapy in patients with asymptomatic BM	Clinical benefit rate
Combination of immune checkpoint inhibitor with radiotherapy					
NCT02097732	Phase II	Ipilimumab	Stereotactic radiosurgery	Ipilimumab induction flowed by stereotactic radiosurgery and ipilimumab in patients with oligo- to asymptomatic BM	Local control rate
NCT02115139	Phase II	Ipilimumab	Stereotactic radiosurgery	Combination of ipilimumab and stereotactic radiosurgery in newly diagnosed BM patients	1 year survival rate
NCT02858869	Pilot	Pembrolizumab	Stereotactic radiosurgery	Combination of pembrolizumab and stereotactic radiosurgery	Proportion of dose limiting toxicities
NCT02716948	Pilot	Nivolumab	Stereotactic radiosurgery	Combination of nivolumab and stereotactic radiosurgery	Incidence of serious adverse events
New therapy approaches					
NCT02681549	Phase II	Pembrolizumab + bevacizumab	None (prior radiotherapy at most 14 days before treatment allowed)	Combination of pembrolizumab + bevacizumab	Brain metastasis response rate
NCT02452294	Phase II	Buparlisib (PI3K Inhibitor)	None	Buparlisib monotherapy in patients with BM not eligible for radiotherapy or surgery	Intracranial disease control rate
NCT01904123	Phase I	WP1066 (STAT3 inhibitor)	None	WP1066 monotherapy	Maximum tolerated dose
NCT02308020	Phase II	Abemaciclib (CDK inhibitor)		Abemaciclib in patients without or stable steroid dosage	Percentage of participants achieving complete response

### Combination of BRAF inhibitors with radiotherapy

The combination of BRAF inhibitors and radiotherapy is subject to an on-going discussion. Questions including the sequencing and dosing are currently investigated in several clinical trials (Table 2). In general, melanoma is considered a radio-resistant tumor. Only small retrospective case series postulate an increased response rate by the combination of Vemurafenib and radiotherapy. This is further supported by preclinical findings suggesting a radio-sensitizing effect of Vemurafenib [45, 61]. On the other hand, increased side effects such as skin symptoms have been reported for the combination, thus potentially limiting the clinical applicability [1, 29]. Based on these data, BRAF inhibitors should probably be paused during the radiotherapy.

### Side effects of BRAF inhibitors in brain metastasis patients

Classical side effects of BRAF inhibitors include gastrointestinal symptoms (diarrhea, nausea, vomiting), skin symptoms (cutaneous squamous cell carcinoma, keratoacanthoma, photosensitivity reaction, hyperkeratosis, rash), fatigue, pyrexia, and elevated liver enzymes [23, 42]. Frequency of cutaneous squamous cell carcinoma and keratoacanthoma is lower in patients treated with the combination of BRAF and MEK inhibitor [30]. No additional and particular no increase in neurological side effects were observed in BM patients, showing that BRAF inhibitors are safe in this particular population [34, 41••].

## Immune checkpoint inhibitors

Immune checkpoint inhibitors have dramatically changed the treatment of metastatic melanoma with a substantial overall survival improvement [24, 57–59]. So far, CTLA4 (cytotoxic T-lymphocyte protein 4; Ipilimumab) and PD-1 (programmed cell death 1; Nivolumab, Pembrolizumab) axis targeting immune checkpoint inhibitors have been approved for the treatment of metastatic melanoma. The anti-tumor effect is mediated by T cells as immune checkpoint inhibitors increase the tumor-specific T cell response [40]. Response assessment might be challenging as pseudoprogression caused by the initial influx of immune cell in the tumor tissue can mimic disease progression but may result in subsequent disease control [72]. Importantly, response patterns to immune checkpoint inhibitors may differ from traditional chemotherapy and other targeted therapies. The period to best response can be longer than with targeted therapies or chemotherapy. Some patients present initially with a stabilization of the disease and delayed tumor shrinkage only after several weeks or months of treatment. The resulting responses may however be long lasting and some patients present with disease stabilization for prolong periods [24, 57, 58]. Long-term follow-up of patients treated in phase II and III trials with ipilimumab reveals a survival plateau of 20% starting from 3 years and extending up to 10 years [62]. The response to PD-1 axis targeting checkpoint inhibitors or the combination of both have shown even more profound responses than Ipilimumab monotherapy, and the long-term follow-up indicates an even higher survival plateau [57, 58].

### Predictive biomarkers

Several potential predictive biomarkers have been postulated for the response to immune checkpoint inhibitors [18]. For ipilimumab, some histological characteristics like high density of tumor infiltrating lymphocytes as well as genetic characteristics including a neo-antigen signature and high mutational load were postulated to have a predictive potential [20, 64, 69•]. Expression of PD-L1 on the tumor cells is a promising predictive biomarker for the response to PD-1 axis targeting immune checkpoint inhibitors. However, the negative predictive value of PD-L1 expression is low, as responses were also observed in a significant fraction of patients without PD-L1 expression [67]. Here, several different antibodies and cut off values were postulated. The probability of response increases with the percentage of PD-L1 expressing cells, with the highest probability in patients with >50% of tumor cells presenting with expression [60, 70, 73]. A high density of tumor infiltrating lymphocytes as well as a similar frequency of PD-L1 expression as compared with extracranial tumor sites has been reported for melanoma BM [7].

### Clinical efficacy of immune checkpoint inhibitors in melanoma brain metastases

It has been of dispute whether immune checkpoint inhibitors can reveal their full therapeutic potential in BM, as these monoclonal antibodies with a large molecular weight are unable to cross the blood brain barrier [8, 9]. However, the mode of action of immunotherapies has to be taken into account, as the T cell priming influenced by immune checkpoint inhibitors may to some extent occur in immunological sites distant from the tumor (e.g., regional lymph nodes). T cells activated in such sites may cross an intact blood brain barrier [52]. Further, the blood brain barrier is disrupted in contrast enhancing BM. Consequently, anti-PD-1 immune checkpoint inhibitors may be able to enter and inhibit the binding of PD-L1 (programmed cell death ligand 1) on tumor cells and the PD-1 receptor on T cells in at least some areas of BM. In line with these considerations, clinical activity of immune checkpoint inhibitor monotherapy was observed in melanoma BM patients. A phase II study on ipilimumab monotherapy in patients with melanoma BM included overall 72 patients in two cohorts. Cohort A (*n* = 51) included asymptomatic patients without corticosteroid treatment and cohort B (*n* = 21) patients with symptoms requiring corticosteroid treatment [39]. Intra- (cohort A: 25%, cohort B: 10%) and extracranial (cohort A: 33%, cohort B: 10%) disease control rate applying adapted response criteria was similar, although a difference was observed between the asymptomatic patients (cohort A) and the symptomatic ones (cohort B). Therefore, corticosteroid treatment might impact the efficacy of ipilimumab therapy either due to the resulting immune suppression or due to the impaired clinical condition of the patients, which is resembled by the need for corticosteroid treatment. Here, data from extracranial metastatic melanoma indicates that corticosteroid treatment for the control of immune related side effects does not alter response probability [21, 25]. In symptomatic BM patients in need for steroid treatment, the disease progression may be too fast to allow enough time for the immune checkpoint inhibitor to develop its full antitumor activity. As PD-1 axis targeting immune checkpoint inhibitors have shown higher response rates in extracranial melanoma, these might also be the more promising agents for melanoma BM patients. So far, however, only little data on the intracranial efficacy of anti-PD-1 immune checkpoint inhibitor monotherapy exists. A phase II trial on pembrolizumab monotherapy in BM patients included different primary histologies including melanoma (*n* = 18) and non-small cell lung cancer (*n* = 18) [19••]. Unfortunately, not all melanoma patients were eligible for response assessment, however 4/14 (28%) patients presented with confirmed intracranial partial response and 2/14 (14%) with stable intracranial disease, resulting in a 42% intracranial disease control rate. Extracranial disease control was 50%, with 4/16 (25%) patients presenting with extracranial partial or complete response and 4/16 (25%) with stable disease. Conclusions have to be drawn with caution due to the limited number of included patients. Nonetheless, observed response rates are promising and currently recruiting trials are further addressing the efficacy of pembrolizumab in patients with melanoma BM (Table 2). No results from BM specific clinical trials, investigating the efficacy of nivolumab treatment in melanoma BM patients, are yet available. However, several case series indicate the safety and efficacy of nivolumab monotherapy [6, 37]. The currently recruiting clinical trials will provide a deeper and more profound insight in the value of immune checkpoint inhibitor therapy in melanoma BM patients (Table 2).

### Combination of immune checkpoint inhibitors with radiotherapy

Combination of radiotherapy and immune checkpoint inhibitors has shown to increase response also in not initially radiated lesion due to the so-called abscopal effect [49, 66]. In theory, antigen release due to cell death caused by the radiation increases the T cell mediated immune response facilitated by the immune checkpoint inhibitor treatment. Therefore, several case series postulate an increased response rate through the combination and sequencing of immune checkpoint inhibitors and stereotactic radiotherapy [16, 26•, 53]. However, definitive conclusions cannot be drawn yet but currently recruiting clinical trials addressing this question in melanoma BM patients will provide further insights (Table 2).

### Combination of immune checkpoint inhibitor and chemotherapy

As previously addressed for the combination with radiotherapy, the combination with chemotherapy is also postulated to increase antigen release and thereby potentiate the efficacy of the immune checkpoint inhibitor therapy. A phase II clinical trial focused on the combination of fotemustine and ipilimumab and included a subcohort of BM patients (*n* = 20). Again a cohort included patients with previous radiotherapy (*n* = 7) and a treatment naïve cohort with asymptomatic BM (*n* = 13). Here, 10/20 (50%) patients achieved intracranial disease control (SD + PR + CR) [15]. As a consequence, the applied combination of fotemustine and ipilimumab is currently studied in subsequent clinical trials (Table 2).

### Combination of two immune checkpoint inhibitors

Combination approaches of ipilimumab and nivolumab were shown to result in significantly improved progression-free survival in patients with metastatic melanoma [31]. However, treatment-related adverse events also increased in the combination arm with grade 3 and 4 events occurring in up to 55% of patients. A small case series of nine patients (6/9 with BM) populates safety of the combination of low dose ipilimumab (1 mg/kg) with pembrolizumab in BM patients as no increase in neurological side effects was observed [27]. Again, several on-going clinical trials are currently addressing this promising combination (Table 2).

### Combination of BRAF inhibitor and immune checkpoint inhibitor

Combination of a BRAF inhibitor, producing fast effective tumor shrinkage, and an immune checkpoint inhibitor with slowly occurring but potentially long-lasting response would be tempting. Unfortunately, initial safety data revealed a marked liver toxicity of the combination [54]. Sequencing with induction of the BRAF inhibitor and switch to the immune checkpoint inhibitor after few weeks might be an interesting strategy, which is currently subject to clinical trials (Table 2) [3].

### Side effects in brain metastasis patients

PD-1 axis targeting immune checkpoint inhibitors are generally better tolerated compared to CTLA-4 immune checkpoint inhibitors [58]. The side effects are mostly caused by the increased immune response and encompass several auto-immune mediated side effects. Most frequent adverse events in patients treated with PD-1 axis targeting immune checkpoint inhibitors are colitis, pneumonitis, skin reactions, and endocrine (thyreoiditis, hypophysitis) adverse events [57, 58]. Similarly, diarrhea, rash, puritus, liver enzyme increase, and endocrine (thyreoiditis, hypophysitis) adverse events are the most common one in ipilimumab-based treatment [24]. The side effect profile among BM patients presented to be not significantly different compared to patients without brain metastases, especially no particular increase in neurological side effects was observed [19••, 39]. Pseudoprogression with resulting symptoms due to the volume increase was occasionally reported for BM patients, especially for the combination of immune checkpoint inhibitors and stereotactic radiosurgery [14, 26•].

## Conclusions

The development of several new systemic treatment options including, BRAF and MEK as well as CTLA-4 and PD-1 targeting immune checkpoint inhibitors have revolutionized the daily practice of melanoma treatment [40]. Early BM-specific clinical studies have postulated comparable intra- and extracranial response rates for this new generation of targeted therapies [19••, 34, 39, 42]. Although encouraging, several important questions on the combination, the sequencing or the concomitant application of radiotherapy remain unanswered. The currently recruiting BM-specific trials are likely to answer some of these questions and give a further direction for the optimal treatment combination in melanoma BM patients (Table 2). Further, the next generation of targeted and immune therapies is in the pipeline. Here, PI3K or CDK inhibition might be promising as well as immune modulating therapies targeting e.g., PD-L1, Lag3 or Tim3 [4, 5, 13, 46]. In conclusion, targeted therapies including BRAF inhibitors and immune checkpoint inhibitors have become an important backbone in the treatment of melanoma BM and the on-going clinical trials will further redefine the clinical practice in this particular patient cohort.
